# Lecture on Dentition and Its Derangements

**Published:** 1861

**Authors:** A. Jacobi

**Affiliations:** Professor of Infantile Pathology and Therapeutics


### LECTURE ON DENTITION AND ITS DERANGEMENTS.

By A. JACOBI, M. D.,
*Professor of Infantile Pathology and Therapeutics.*

"*Anatomy and Physiology of Mucous Membrane in General—Nature of its Secretion—General Pathology of Mucous Membrane—General Etiology—Primary and Secondary Nature of Diseases—External Injuries—Cold—Atmospheric and Epidemic Influences—Constitutional Poisons—Contiguity and Sympathy—Different Forms and Symptoms.*

I have several times alluded to certain qualities and actions of the mucous membranes in general. But you will better understand many of the foregoing remarks, after following me in the investigation of several anatomical and physiological facts, concerning both the structure and function of the mucous membranes. And certainly, they are deserving of every attention that can be bestowed upon them, not only on account of the large surface they cover, but also in consequence of their physiological and pathological importance. As the cutis forms the external integument of the body, so does the mucous membranes form the internal covering of any and all the organs. Thus, we find them all over the respiratory, digestive, uropoietic, and sexual organs, and in all those isolated cavities, like the maxillary and frontal ones, which are connected with the larger ones by narrow ducts; further in the glands, the affections of which are either genuine and primary, or continuous only of a morbid process on a mucous membrane, and their ducts; the conjunctivæ, the external ear, and the galactiferous ducts of the female breast.

The mucous membrane of an organ or a system is not confined to certain limits. Except on the lips, on the external ear, and other localities where we distinctly perceive the gradual replacement of epidermis and cutis by mucous membrane of this organ or system finds its end, and that of the other commences. Thus, there is no boundary between the mucous membranes of the digestive and the respiratory organs, nor any between that of the stomach and duodenum. Their internal structure is alike, and therefore I deem it more proper henceforth to speak rather of mucous membrane, than of mucous membranes. Its uniform layer consists of dense connective tissues, intermixed with blood-vessels and elastic fibres, in its deeper laminæ with muscular fibres also, and is covered with several layers of epithelial scales, which are readily thrown off and renewed; they may, however, be accumulated in yellowish, brownish, or black masses. Beside the blood-vessels there are lymphatic vessels, and the smallest ramifications of nerves, which are particularly found in the papillary prominences. They are either the last ends of a cerebral or spinal, or of the sympathetic nerve; the peculiar actions of these several nerves determining the functions of the locality in which they spread. The mucous membrane, influenced by the cerebro-spinal system, is more sensitive, as a general rule, than such localities in which the power of the sympathetic prevails. Thus, pain depends on the seat of the affection just as well as on its acuteness; the degrees of temperature are discerned by the pharynx, but not by the stomach or intestine; urine produces no pain whatever on the mucous membrane of the urinary bladder and urethra, but very much so on that of the conjunctivæ; and often irritations meeting a mucous membrane effect no pain or other local disturbance, but sympathetic sensations, like coughing or sneezing.

The functions of the mucous membrane are both various and important. It takes a prominent part in assimilation and sanguification, and therefore suffers in all general, and all those local diseases that in any way influence the general condition of the organism. They are frequently first affected in a large number of diseases, many of which are primary; for the immense extension of mucous membrane, increased by indentations, villi, glands and glandular ducts, and papilæ, is such that morbid processes may easily take place in one part or another. The influence of the diseased mucous membrane is, moreover, as great as its effects are frequent; the vital importance of the membrane itself, the legions of nerve ramifications in its tissue, and the contiguity and rapidly developed consecutive affections of the mucous membrane contributing to the same result. The occurrence of œdema of the glottis in catarrh of the pharynx or larynx, or of collateral œdema of the vocal cords in diphtheritic inflammation of the larynx, are distinct and much dreaded proofs of this fact.

In its normal condition, the mucous membrane exhibits a peculiar tough, whitish or clear, more or less transparent, alkaline secretion, called mucus. It contains mostly epithelial scales, more or less transformed, of every variety; pavement, cylindrical, and vibrating, the latter without its cilia; further, round granulated cells with one or more nuclei, and a clear transparent liquid. Epithelium, mucus, and pus are found combined in many instances of secretion on the mucus membrane, the three various forms being, in this locality, but three different stages of the transforming epithelium. Under favorable circumstances, the mucous membrane forms puriform elements anywhere, but there is some difference in the process. The purulent mucus of the intestine contains very seldom puriform elements, that is, pus cells, except in cases of genuine ulceration; the same result is found on examination of the purulent mucus of the uterus and tubes. But no ulceration is required in the mucus membrane of the bladder or urethra to count immense numbers of pus cells in the puriform secretion of chronic vesicular catarrh and gonorrhœa. This difference depends on anatomical reasons. The intestine, uterus, and Fallopian tubes have cylindrical epithelium only, bladder and uretha, however, pavement epithelium. The mucus membrane will develop the more pus cells without the presence of real ulceration in proportion to the amount of pavement epithelium by which it is covered. Purulent though the secretion of other parts of the mucus membrane will look, it contains frequently nothing but cylindrical epithelium. The angular shape of the pavement epithelium enables it to form a cohering covering, which is not thrown off so easily as the round pus or mucous cells; thus the lower layers have full time to develop into mucous cells; which results being obtained, the whole mass is thrown off by either the pressure of the subjacent new layers, or the influence of the thin and less cohesive transudation from the blood-vessels, which, in its turn, forms another important element of the secretion. Whether it has a more important part than to be one of its elements, whether, for instance, from its substance cells may be developed, or whether the cells are under all circumstances but the later stages in the development of epithelial scales, is still an open question. This is certain, that what is called mucus is by no means always the same liquid, no more so than that the secretion of the external skin is alike on every locality of its surface. Its reaction is acid in the stomach, alkaline in the mouth and intestinal canal; a mucous substance is secreted from the parenchymatous substance of some organs, without the presence of cells; there are pathological liquids, as colloid, very similar to mucus; there is the substanc called after the name of Wharton, in the umbilical cord of the fœtus and newly born, the cellular development of which cannot be traced; and nevertheless this "gelatinous connective tissue" is transformed into mucus. Thus, from an anatomical point of view, the secretion of the mucous membrane is not a uniform substance; neither is it uniform as to chemical composition. I have stated that its reaction differs according to localities. It frequently contains albumen, some little fatty substance, extractive matters, and some mineral elements, as chloride of alkalines and phosphates of earths. These mineral elements belong to the mucine, which is a nitrogenous, albuminous substance, swelling in water, but not dissolved by it, and to which the mucus owes its tough nature. Its chemical reaction differs according to its percentage of minerals, combination with other poisonous substances, or its own peculiar modifications. This difference is easily explained by the fact, that it is not performed in the blood and thrown on the surface, but is a production of the mucous membrane itself. Thus its constitution depends on the amount of follicles, epithelial scales, papilæ, and on the character of the epithelium; no matter whether it is formed directly from the epithelium undergoing its final changes, or from transudation through the walls of the capillary vessels.

In regard to the diseases of the mucous membrane, I have already stated both their frequency of occurrence and their proclivities for complication. Their tendency to sickness is, however, not uniform; individuality and age belonging to those influences which are most apt to modify the alterations taking place in their tissue or secretion. Affections of the mucous membrane are very rare in fœtal life, because of the absence of both mechanical injuries and functional disorders. In infantile age the mucous membrane reaches its greatest importance, new influences acting upon it and calling into life new functions, especially the normal state of injection, which is very considerable indeed. A very common alteration taking place in the mucous membrane is mollification; plastic exudation, hæmorrhage, suppuration, and ulceration being very rare in the first year of life. After this time exudation processes are more numerous, especially fibrinous exudations are not unfrequent. This predisposition of early age to contract diseases of the mucous membrane, is afterward decreasing, is not very common in advanced age, until in senile age it is rather increasing.

A number of diseases of the mucous membrane in early age are of a primary nature, and many of them result from direct local injuries. It is a singular fact, however, that thorough and deep local injuries, cuts and wounds of any kind of the mucous membrane, dangerous though they look, are attended with very little danger in the majority of cases; they will generally heal readily and lose nothing of their merely local character. Thus foreign bodies entering the substance, combustion destroying the structure of the mucous membrane, although sometimes among the causes of a disease, will not so frequently give rise to a severe affection, as a less serious injury often repeated. Animal or vegetable parasites, and indigestible food, will therefore, as their influence extends over a longer period, although their sudden insult is often but inconsiderable, be among the most frequent diseases of the mucous membrane of the digestive organs. Another very important and frequent cause of diseases of the mucous membrane is refrigeration. We are entitled to state this as a fact, although we do not know whether cold acts by the suppression of cutaneous secretion alone, or by some influence on the peripheric cutaneous nerves and reflex action alone; or by both. It is, however, a fact that especially the mucous membrane of both the respiratory and digestive organs is very subject to the influence of cold, together with the other causes of disease depending on the general condition of the atmosphere, and the changes and general influences of season, of epidemics and endemics.

These latter are of great importance in the etiology of the affections of the mucous membrane in early age; for we know that not only malarious influences and animal effluvia will readily act on the impressible infantile organism, but the constitutional and contagious poisons are mostly observed to produce their peculiar forms of disease in infantile age. Thus children are the majority of patients suffering from eruptive fevers; scarlatina, measles, and diphtheria mostly attacking the infantile organism. And here it is important to state that a peculiar part of the mucous membrane has always a tendency to be affected by a peculiar constitutional poison, both in early and advanced age. Thus diphtheria, scarlatina, syphilis and mercurialism show a predilection for the mucous membrane of the mouth and pharynx, typhus for the ileum, dysentery for the colon, measles and iodism for conjunctivæ and nose. All such affections, although common to every age, are mostly found in the infantile period, the modes of propagation and transmission being eminently distinct at this period of life.

I have frequently alluded to many cases of secondary affections of the mucous membrane; they are the usual results of either local propagation in the continuity of tissue, or of sympathetic spreading. We know from general pathology that there is a direct connection between cutis and mucous membrane, scalp and nose, mamma and uterus, urethra and testicles, and stomach and brain; we need not be astonished then, that there is a contemporaneous affection sometimes of the mucous membrane of the nose and the lungs, the larynx and trachea being free from disease; or of the stomach and colon, the small intestines being not at all affected. And the spreading of affections of the mucous membrane on continuous tissue is so very general, that labular pneumonia, for instance, is in all cases recognized as the termination of a catarrh of the bronchi; and a protracted catarrh of the colon with ulceration of the follicles is known to be a usual consequence of catarrh of the small intestines. Nor is the topical propagation of affections of the cutis over the adjoining mucous membrane an exception, but the rule. The transmission of diphtheritic and other processes of the external integuments of the lips, anus, and pudenda majora, on the mouth, rectum, and vaginia, are frequently observed.

Thus it appears that nothing is more natural than a universal or wide-spreading hyperæmia, changes in both quantity and quality of secretions, rupture of blood-vessels, and even neoplasms. The alterations observed in the secretions are frequently more important in relation to post-mortem epicrises, than the anatomical change of the tissue itself; for you have learned already, from a previous lecture, that not unfrequently no anatomical trace is detected in patients who have died from, or with, hyperæmia of the brain, pharynx, intestines, or cutis. The abnormal secretions are therefore as important elements in regard to the results of post-mortem examination, as they again are ready causes of renewed attacks, from the local irritation depending on their presence on the membrane. The prognosis, therefore, depends greatly on their nature and amount, and frequently as much on them as on the structure of the membrane, its epithelium, follicles, or papillæ. To a great extent they also influence the symptoms, among which functional disorders and anomalous secretions are always prominent. Pain is sometimes observed, but it is frequently indistinct and obscure. Of more importance than the latter, however, are some indirect symptoms, of which reflected motions, and even muscular paralysis, are frequently met with. Thus, sneezing, coughing, vomiting, and tenesmus are brought on. Disorders in neighboring or distant parts are effected by the suppression of secretion and injection of the tissues; topical spreading on the subjacent submucous tissue, as in œdema of the glottis, and retropharyngeal abscess; and participation of the whole system.

After these general remarks you are enabled to trace a direct connection between even the slightest causes and severe affections. I have taken particular pains, in former lectures, to present for your inspection a number of affections which, severe though they be, owe their origin frequently to a comparatively insignificant cause. The greatest stress has been laid by me, further, on the large number of slight or unimportant causes giving rise to affections of the mucous membrane. That in some cases an abnormal process of dentition will prove a source of evil, I do not deny; but from many previous remarks, and from comparison with other causes of diseases, you have arrived at the conclusion that the vast majority of diseases of the mucous membrane allow of another explanation than the blind assumption of the culpability of a physiological process. The great progress of pathalogical anatomy and differential diagnosis ought not to be lost on us. The period, where the diseases of small children consisted in dentition, of advanced ones in worms and scrofula, of adults in rheumatism, scrofula, and syphilis, is past. With sound principles in pathology, and a correct knowledge of pathological anatomy and differential diagnosis, all the different and numerous affections of the mucous membrane; simple injection, with or without extravasation; acute hyperæmia, with increase and alteration of the secretion, and follicular swellings; acute serous or bloody exudations, with more or less severe symptoms; pseudo-membranous deposits of epidemic, cyphilitic, or mercurial character; purulent discharges; ichorous decomposition; chronic alteration of both vascularization and secretion; hæmorrhage; œdema; hypertrophy and whatever changes we have learned to take place in the mucous membrane of all the organs that have been submitted to your attention in previous lectures—will no longer present to you the difficulties of bygone times, nor urge upon you the necessity of resorting to an obscure, generally erroneous and improbable, and almost always unproven explana tion.—*Amer. Med. Times.*

